# Maternal and perinatal outcomes of hypertensive disorders in pregnancy: Insights from the National Hospital of Obstetrics and Gynecology in Vietnam

**DOI:** 10.1371/journal.pone.0297302

**Published:** 2024-01-31

**Authors:** Nguyen Thi Huyen Anh, Nguyen Manh Thang, Truong Thanh Huong

**Affiliations:** 1 Hanoi Medical University, Hanoi, Vietnam; 2 National Hospital of Obstetrics and Gynecology, Hanoi, Vietnam; 3 Phenikaa University, Hanoi, Vietnam; University of Cambridge, UNITED KINGDOM

## Abstract

**Introduction:**

Hypertension is the common disorder encountered during pregnancy, complicating 5% to 10% of all pregnancies. Hypertensive disorders in pregnancy (HDP) are also a leading cause of maternal and perinatal morbidity and mortality. The majority of feto-maternal complications due to HPD have occurred in the low- and middle-income countries. However, few studies have been done to assess the feto-maternal outcomes and the predictors of adverse perinatal outcome among women with HDP in these countries.

**Methods:**

A prospective cohort study was conducted on women with HDP who were delivered at National Hospital of Obstetrics and Gynecology, Vietnam from March 2023 to July 2023. Socio-demographic and obstetrics characteristics, and feto-maternal outcomes were obtained by trained study staff from interviews and medical records. Statistical analysis was performed using SPSS version 26.0. Bivariate and multiple logistic regressions were done to determine factors associated with adverse perinatal outcome. A 95% confidence interval not including 1 was considered statically significant.

**Results:**

A total of 255 women with HDP were enrolled. Regarding adverse maternal outcomes, HELLP syndrome (3.9%), placental abruption (1.6%), and eclampsia (1.2%) were three most common complications. There was no maternal death associated with HDP. The most common perinatal complication was preterm delivery developed in 160 (62.7%) of neonates. Eight stillbirths (3.1%) were recorded whereas the perinatal mortality was 6.3%. On bivariate logistic regression, variables such as residence, type of HDP, highest systolic BP, highest diastolic BP, platelet count, severity symptoms, and birth weight were found to be associated with adverse perinatal outcome. On multiple logistic regression, highest diastolic BP, severity symptoms, and birth weight were found to be independent predictors of adverse perinatal outcome.

**Conclusion:**

Our study showed lower prevalence of stillbirth, perinatal mortality, and maternal complication compared to some previous studies. Regular antenatal care and early detection of abnormal signs during pregnancy help to devise an appropriate monitoring and treatment strategies for each women with HDP.

## Introduction

Hypertension in pregnancy is defined as a systolic pressure ≥ 140 mmHg and/or a diastolic pressure ≥ 90 mmHg [1]. Hypertension is the common disorder encountered during pregnancy, complicating 5% to 10% of all pregnancies [2]. Hypertensive disorders in pregnancy (HDP) include preeclampsia–eclampsia, chronic hypertension, gestational hypertension, and chronic hypertension with superimposed preeclampsia [1]. Globally, HDP remain the leading causes of maternal and perinatal morbidity and mortality, with an estimated number of 30,000 maternal deaths and 500,000 perinatal deaths each year [3]. HDP are associated with adverse perinatal outcomes like intrauterine growth restriction, prematurity, preterm delivery, perinatal asphyxia, stillbirths and neonatal mortality [4, 5]. HDP also result in an increased risk of adverse maternal outcomes including HELLP syndrome, placental abruption, stroke, renal damage, hepatic injury, pulmonary edema, and death [6, 7]. HELLP is a syndrome characterized by haemolysis, elevated liver enzymes, and low platelets. HELLP syndrome occurs in about 0.5% to 0.9% of all pregnancies and complicates 10% to 20% of women with severe preeclampsia. HELLP syndrome is one of the common cause of maternal and fetal mortality among women with HDP [8].

The majority of feto-maternal complications of HPD have occurred in the low- and middle-income countries due to lack of healthcare service as well as poor quality of maternal and neonatal care [9, 10]. WHO estimated that the incidence of preeclampsia was 7 times higher in low- and middle-income countries than in high-income countries, and the risk of pregnant women in a low-income country dying of pre-eclampsia/eclampsia was 300 times higher than those in a high-income country [11]. However, few studies have been done to assess the feto-maternal outcomes and identify the predictors of adverse perinatal outcome among women with HDP in these countries. To the best of our knowledge, there are few hospital-based reports on HDP in Vietnam and most studies only focus on preeclampsia that is a common type of HDP. In this context, exploring feto-maternal outcomes of HDP will be important to give useful information for healthcare providers and policy makers to design appropriate interventions. Therefore, our study aimed to assess the feto-maternal outcomes and determine factors associated with adverse perinatal outcome among women with HDP in Vietnam.

## Material and methods

### Study design and setting

We conducted a prospective cohort study of women with HDP who were delivered at National Hospital of Obstetrics and Gynecology from March 8^th^ 2023 to July 31^st^ 2023. National Hospital of Obstetrics and Gynecology is a government referral hospital in Hanoi, the capital of Vietnam. It has served since 1955. This hospital has 1470 health professionals of which 199 are obstetricians, gynecologists, and neonatologists, and 688 are midwives and nurses. It has a total of 1000 Obstetrics and Gynecology ward beds and 20 delivery coaches.

### Participants

Our study population included all women with HDP who were 18 years or above and were delivered after 28 weeks of gestation at National Hospital of Obstetrics and Gynecology. Excluded from the study were women with HDP who declined to participate in the study and those with other medical disorders like renal, hepatic, cardiovascular, neuronal or endocrine disorders. Women who met the inclusion criteria and consented to the study were enrolled within 24 hours of delivery and followed up to 12 weeks postpartum. This study was part of a prospective cohort study to examine the magnitude of persistent hypertension at 12 weeks postpartum after HDP. Mothers were interviewed within 24 hours of delivery, at 3 days, 7 days, 6 weeks and 12 weeks postpartum by trained study staff.

### Variables and data sources

Data was collected using a structured questionnaire developed by the research authors after reviewing literatures. The data collection involved daily identification of all the women with HDP who were delivered within the previous 24 hours in the hospital. The study protocol was explained to each individual in detail and those who gave written informed consent were included in the study. After receiving written informed consent, the socio-demographic and obstetrics characteristics, and the feto-maternal outcomes were obtained by trained study staff during the participant’s hospital stay or within seven days of delivery. Their medical records were also reviewed to determine the maternal and perinatal outcomes of their pregnancies.

In this study, we defined hypertension as two blood pressure (BP) readings with either a systolic BP ≥ 140 or a diastolic BP ≥ 90 mmHg measured 4 hours apart. Hypertensive disorders in pregnancy were classified as preeclampsia—eclampsia, gestational hypertension, chronic hypertension and preeclampsia superimposed on chronic hypertension. Preeclampsia was characterized by a blood pressure of 140/90 mmHg or greater after 20 weeks’ gestation in a woman with previously normal blood pressure and who had proteinuria. In the absence of proteinuria, preeclampsia was diagnosed as hypertension in association with thrombocytopenia, impaired liver function, the new development of renal insufficiency, pulmonary edema, or new-onset cerebral or visual disturbances. Eclampsia was defined as seizures that cannot be attributable to other causes, in a woman with preeclampsia. Gestational hypertension was blood pressure elevation after 20 weeks of gestation in the absence of proteinuria or the aforementioned systemic findings. Chronic hypertension was hypertension that predated pregnancy or diagnosed before 20 weeks of pregnancy, and superimposed preeclampsia was chronic hypertension in association with preeclampsia [1].

Adverse perinatal outcome was defined as a composite of one or more of the following: intrauterine growth restriction, preterm delivery, perinatal asphyxia, stillbirth, and perinatal death. Intrauterine growth restriction was defined as a birth weight of newborn below the tenth percentile of weight distribution at the specified gestational age of a pregnancy. Preterm delivery was a birth of baby occurring after 22 completed weeks but before 37 completed weeks of gestation. Perinatal asphyxia was defined as a neonatal condition defined by five minute APGAR score of less than seven. Stillbirth was death prior to the complete expulsion or extraction from its mother of a product of conception after 28 weeks of pregnancy. Perinatal mortality was stillbirths and newborn deaths within the first seven days of delivery.

### Sample size and power

The sample size was based on the primary cohort study examining persistent hypertension. For this sub-study, sample size and power were therefore not determined a priori.

### Data analysis

The analysis of the data was performed using SPSS version 26.0 software. Descriptive statistics were used to calculate percentages. The results were presented in numbers and percentages. Bivariate and multiple logistic regression were done to determine factors associated with adverse perinatal outcome. Variables which did not show statistical significance in the bivariate analysis were excluded from the multivariate analysis. A statistically significant association was considered when the odds ratio (OR) 95% confidence interval did not include the number 1.

### Ethical consideration

Ethical approval was obtained from Institutional Review Board for Ethics in Biomedical Research–Hanoi Medical University (Approval Number: 820/GCN-HĐĐĐNCYSH-ĐHYHN). All the study participants gave a written informed consent prior to the commencement of the study.

## Results

### Socio-demographic and obstetrics characteristics of participants

A total of 255 women with HDP were included in the study during the period from March to July 2023. Most of the pregnant women, 231 (90.6%) were urban residents. The average age of mothers was 31.49 ± 6.45 years with a minimum of 18 years and a maximum of 53 years. 108 (42.4%) of the pregnant women were primigravida. More than half of mothers (54.1%) were nulliparous. Most of the pregnant women (89.0%) had no previous history of HDP. Gestational diabetes mellitus accounted for over 17% of the patients. The rate of multiple pregnancy in our study population was 22.4%. Of all the HDP cases, 182 (71.4%) were preeclampsia–eclampsia, 51 (20%) were gestational hypertension, 13 (5.1%) were superimposed preeclampsia, and 9 (3.5%) were chronic hypertension. 35 (13.7%) of women with HDP presented to the hospital with headache as the chief complaint. Rare symptoms that were observed from some of the patients were blurring of vision (3.5%) and epigastric pain (0.4%) (Table 1).

**Table 1:** Characteristic of women with HDP at National Hospital of Obstetrics and Gynecology, Vietnam, 2023.

Variables	N	%
Residence		
Urban	231	90.6
Rural	24	9.4
Maternal age		
< 25	47	18.4
25–30	75	29.4
> 30	133	52.2
Gravidity		
Primigravida	108	42.4
Multigravida	147	57.6
Parity		
Primiparity	138	54.1
Multiparity	117	45.9
Smoking		
Yes	80	31.4
No	175	68.6
Previous history of HDP		
Yes	28	11.0
No	227	89.0
Gestational diabetes mellitus		
Yes	45	17.6
No	210	82.4
Multiple pregnancy		
Yes	57	22.4
No	198	77.6
Type of HDP		
Chronic hypertension	9	3.5
Gestational hypertension	51	20.0
Preeclampsia–Eclampsia	182	71.4
Superimposed preeclampsia	13	5.1
Severity symptoms		
Headache	35	13.7
Epigastric pain	1	0.4
Blurring of vision	9	3.5
Highest systolic BP (mmHg)		
< 140	49	19.2
140–159	88	34.5
≥ 160	118	46.3
Highest diastolic BP (mmHg)		
< 90	86	33.7
90–109	128	50.2
≥ 110	41	16.1
Platelet count (G/l)		
< 100	17	6.7
≥ 100	238	93.3
Proteinuria (g/l)		
< 2	147	57.6
≥ 2	108	42.4
AST (UI/l)		
< 70	237	92.9
≥ 70	18	7.1
ALT (UI/l)		
< 70	236	92.5
≥ 70	19	7.5
Creatinine (mg/dl)		
< 1,1	240	94.1
≥ 1,1	15	5.9
Gestational age (weeks)		
28–33	81	31.8
34–36	79	31.0
≥ 37	95	37.3
Mode of delivery		
Spontaneous vaginal delivery	18	7.1
Assisted vaginal delivery	1	0.4
Cesarean section	236	92.5
Birth weight (g)		
< 2500	153	60.0
≥ 2500	102	40.0
Maternal adverse outcomes		
Elampsia	3	1.2
Placental abruption	4	1.6
Pulmonary edema	1	0.4
HELLP syndrome	10	3.9
Death	0	0
Perinatal adverse outcomes		
Intrauterine growth restriction	63	24.7
Preterm delivery	160	62.7
Perinatal asphyxia	26	10.2
Stillbirth	8	3.1
Perinatal death	16	6.3

A total of 118 (46.3%) had highest systolic BP of 160 mmHg or more and 41 (16.1%) of them had highest diastolic BP of 110 mmHg or more. Thrombocytopenia (platelet count < 100 G/l) was observed in 6.7% of mothers. Over 57% of the patients had proteinuria ≥ 2 g/l. 15 (5.9%) of the pregnant woman developed acute renal failure manifested by the creatinine level of at least 1.1 mg/dl. Impaired liver function manifested by the elevated blood concentrations of liver transaminases to twice normal concentration was seen in more than 7% of mothers. The proportion of preterm delivery was 62.7% whereas low birth weight was 60%. The most common mode of delivery was Caesarean section (92.5%) followed by Spontaneous vaginal delivery (7.1%) and Assisted vaginal delivery (0.4%) (Table 1).

### Feto-maternal outcomes of hypertensive disorders in pregnancy

Regarding adverse maternal outcomes, HELLP syndrome (3.9%), placental abruption (1.6%), and eclampsia (1.2%) were three most common complications. Only 1 (0.4%) of the pregnant women developed pulmonary edema. There was no maternal death reported during the study period. Regarding adverse perinatal outcomes, the study findings showed that the most common perinatal complication was preterm delivery developed in 160 (62.7%) of neonates. The proportion of intrauterine growth restriction was 24.7% among all neonates delivered from hypertensive mothers. APGAR score less than 7 in the 5th minute accounted for 10.2% of neonates. Eight stillbirths (3.1%) were recorded whereas the perinatal mortality was 6.3% (Table 1).

### Factors associated with adverse perinatal outcome

On bivariate logistic regression, variables such as residence, type of HDP, highest systolic BP, highest diastolic BP, platelet count, severity symptoms, and birth weight were found to be associated with adverse perinatal outcome. On multiple logistic regression, the independent predictors of adverse perinatal outcome were highest diastolic BP ≥ 110 mmHg (AOR = 4.33, 95%CI = 1.46–12.86), severity symptoms (AOR = 17.53, 95%CI = 1.54–199.88), and birth weight < 2500 g (AOR = 56.68, 95%CI = 20.47–156.88) (Table 2).

**Table 2:** Bivariate and multiple logistic regression of adverse perinatal outcome of HDP at National Hospital of Obstetrics and Gynecology, Vietnam, 2023.

Variables	Adverse perinatal outcome		COR (95% CI)	AOR (95% CI)
	No n (%)	Yes n (%)		
Residence				
Urban	78 (33.8)	153 (66.2)	1	1
Rural	3 (12.5)	21 (87.5)	3.57 (1.03–12.33)	5.24 (0.77–35.92)
Type of HDP				
CH	5 (55.6)	4 (44.4)	1.14 (0.27–4.77)	0.29 (0.036–2.29)
GH	30 (58.8)	21 (41.2)	1	1
PE	44 (24.2)	138 (75.8)	4.48 (2.33–8.61)	1.14 (0.43–3.04)
PE/CH	2 (15.4)	11 (84.6)	7.86 (1.58–39.17)	1.80 (0.12–26.38)
Highest systolic BP (mmHg)				
< 140	20 (40.8)	29 (59.2)	1	1
140–159	33 (37.5)	55 (62.5)	1.15 (0.56–2.35)	1.29 (0.46–3.61)
≥ 160	28 (23.7)	90 (76.3)	2.22 (1.09–4.51)	1.76 (0.47–6.69)
Highest diastolic BP (mmHg)				
< 90	43 (50.0)	43 (50.0)	1	**1**
90–109	32 (25.0)	96 (75.0)	3.00 (1.68–5.37)	**3.02 (0.58–15.72)**
≥ 110	6 (14.6)	35 (85.4)	5.83 (2.23–15.29)	**4.33 (1.46–12.86)**
Platelet count (G/l)				
< 100	1 (5.9)	16 (94.1)	8.10 (1.06–62.19)	7.81 (0.44–138.22)
≥ 100	80 (33.6)	158 (66.4)	1	1
Severity symptoms				
Yes	1 (2.6)	37 (97.4)	21.61 (2.91–160.50)	**17.53 (1.54–199.88)**
No	80 (36.9)	137 (63.1)	1	**1**
Birth weight (g)				
< 2500	7 (4.6)	146 (95.4)	55.12 (23.00–132.12)	**56.68 (20.47–156.88)**
≥ 2500	74 (72.5)	28 (27.5)	1	**1**

CH: Chronic hypertension, GH: Gestational hypertension, PE: Preeclampsia–Eclampsia, BP: Blood pressure, COR: Crude Odds Ratio, AOR: Adjusted Odds Ratio, CI: Confidence Interval.

## Discussion

This study aimed to assess the feto-maternal outcomes and determine factors associated with adverse perinatal outcome among women with HDP who gave birth at National Hospital of Obstetrics and Gynecology from March 2023 to July 2023. In our study, the prevalence of low birth weight and preterm birth were 60% and 62.7%, respectively. The present prevalences were higher than some studies in Northwest Tigray–Ethiopia (36.8% vs 36.8%) [12], India (40.3%) [13], Uganda (37.1% vs 55.3%) [14], and Addis Ababa–Ethiopia (44.2% vs 32.8%) [15]. This could be attributed partly to the fact that gestational age at termination of pregnancy in our study was earlier than 4 previous studies. It is, however, important to emphasize that the stillbirth rate and perinatal mortality rate in our study were lower than the reports from Ethiopia [12, 15], India [13], and Uganda [14]. Moreover, the prevalence of adverse maternal outcomes including placental abruption (1.6%), pulmonary edema (0.4%), eclampsia (1.2%), and HELLP syndrome (3.9%) in this study were also lower as compared to 4 previous studies. It is worth mentioning that there was no maternal death associated with HDP at National Hospital of Obstetrics and Gynecology during the study period. These findings indicated that early detection and stringent interventions including termination of the pregnancy in severe HDP could contribute to reduce stillbirth rate and perinatal mortality rate, as well as prevent maternal complications. Furthermore, a decrease in adverse maternal and perinatal outcome in this study could be due to the fact that our hospital had improved care facilities, well-trained obstetricians and neonatologists, flexible referral system to the hospital from primary health centers, and appropriate management strategy for antenatal, intrapartum, and postnatal care. Our hospital also gave services for 24 hours a day and 7 days a week. WHO study showed that the availability of basic and comprehensive Emergency Obstetric Care 24 hours per day and 7 days per week in conjunction with a functioning referral system play an important role in preventing most maternal deaths with direct causes [16].

The findings of this study showed that those who were referred from the rural resident had more adverse perinatal outcome as compared to those with mothers from urban. It is consistent with a retrospective study done in Ethiopia [17] and a population-based cohort study done in Canada [18]. This might be related to delay in reaching the facility, poor-quality medical equipment, lack of antenatal care and postpartum follow-up, and weak referral linkage in rural areas. In this study, it was found that perinatal complication respectively four and eight times higher in preeclampsia–eclampsia and superimposed preeclampsia than in gestational hypertension. This is in agreement with a study conducted in Addis Ababa [19] and Wolaita Zone [20]. Previous study proved that the prevalence of low birth weight, preterm delivery, and low Apgar at the first minute and 5th minute were statistically significant association with the severity of HDP [19]. Preeclampsia–eclampsia can lead to higher frequency of neonatal respiratory distress, and increased frequency of admission to neonatal intensive care unit [21].

Regarding to characteristics of HDP, the binary logistic regression analysis demonstrated that highest systolic BP, highest diastolic BP, platelet count, and severity symptoms were predictors of adverse perinatal outcome. This finding is similar with two studies done in Ethiopia [12, 22]. An increase in the risk of perinatal complication with an increase in blood pressure was observed in this study. This might be due to that high blood pressure reduces low normal uteroplacental blood flow, which can affect the well-being of fetuses. Significantly increased risk of perinatal complication were also observed among women with severity symptoms (headache, epigastric pain, blurring of vision) and thrombocytopenia (< 100 G/l). This is probably because the presence of severity symptoms and thrombocytopenia indicates that pregnant women were likely to develop more severe forms of HDP such as eclampsia or HELLP syndrome. These severe forms of HDP are usually complicated by placental abruption, disseminated intravascular coagulation, fetal growth restriction, respiratory distress syndrome or neonatal death due to extreme prematurity [23, 24].

On multiple logistic regression, birth weight was found to be the strongest independent predictor of adverse perinatal outcome. The current study showed that mothers with low birth weight at birth had 56.7 times higher risk of adverse perinatal outcome as compared to those with normal birth weight. This finding is in line with a study conducted by WHO [25], and two previous studies done in Ethiopia [22] and Uganda [14]. It might be related to the scientific fact that HDP is associated with intrauterine growth restriction and preterm deliveries [4]. The transition from intrauterine to extrauterine life requires a rapid adaptation of multiple organ systems so the immaturity of fetal system can cause failure to adaptation of extrauterine life [26]. Furthermore, some previous studies indicated that low birth weight was an important determinant of perinatal survival and intensive care admission [27, 28].

The limitation of our study was the short duration as well as the relatively small number of participants involved. In addition, this study was conducted in a single center, which means that the results may not be representative of the general Vietnamese population. However, the findings from the present study will generate a broad idea about the maternal and perinatal heath problems of HDP in Vietnam and other Southeast Asia countries with similar settings. Multicenter longitudinal studies with well-designed methodology and larger sample size are necessary in the future to expand the research scope, and more importantly, to better inform the optimal management and treatment strategies for women with HDP in Vietnam.

## Conclusion

This study showed lower prevalence of stillbirth, perinatal mortality, and maternal complication compared to some previous studies. Such a low prevalence can be expected in a referral hospital where emergency obstetric and essential newborn care are available. The findings of our study also revealed that rural residence, preeclampsia–eclampsia and superimposed preeclampsia, high BP, thrombocytopenia, the presence of severity symptoms, and low birth weight were predictors of adverse perinatal outcome. Therefore, regular antenatal care and early detection of abnormal signs during pregnancy (hypertension, thrombocytopenia, severity symptoms) help to devise an appropriate monitoring and treatment strategies for each women with HDP.

## Supporting information

S1 Dataset (XLSX)
